# Mastering your fellowship

**DOI:** 10.4102/safp.v63i1.5398

**Published:** 2021-10-25

**Authors:** Mergan Naidoo, Klaus von Pressentin, Tasleem Ras, Ts’epo Motsohi

**Affiliations:** 1 Department of Family Medicine, College of Health Sciences, University of KwaZulu-Natal, Durban, South Africa; 2 Division of Family Medicine, Faculty of Medicine, University of Cape Town, Cape Town, South Africa; 3 Department of Family and Emergency Medicine, Faculty of Health Sciences, Stellenbosch University, Cape Town, South Africa

**Keywords:** family physicians, FCFP (SA) examination, family medicine registrars, postgraduate training, national exit examination

## Abstract

The series, ‘Mastering your Fellowship’, provides examples of the question format encountered in the written and clinical examinations, Part A of the Fellowship of the College of Family Physicians of South Africa (FCFP SA) examination. The series is aimed at helping family medicine registrars to prepare for this examination.

## Introduction

This section in the *South African Family Practice* journal is aimed at helping registrars to prepare for the Fellowship of the College of Family Physicians of South Africa (FCFP SA) Final Part A examination and will provide examples of the question formats encountered in the written examination: multiple choice question (MCQ) in the form of single best answer (SBA – Type A) and/or extended matching question (EMQ – Type R); short answer question (SAQ), questions based on the Critical Reading of a journal (evidence-based medicine) and an example of an objectively structured clinical examination (OSCE) question. Each of these question types is presented based on the College of Family Physicians blueprint and the key learning outcomes of the FCFP programme. The MCQs will be based on the 10 clinical domains of family medicine, the SAQs will be aligned with the five national unit standards and the critical reading section will include evidence-based medicine and primary care research methods.

This month’s edition is based on unit standard 1 (critically reviewing new evidence and applying the evidence in practice, principles of self-care and leading a clinical governance team) and unit standard 2 (evaluating and managing a patient according to the bio-psycho-social approach). The domain covered in this edition is Women’s Health.

Please visit the Colleges of Medicine website for guidelines on the Fellowship examination: https://www.cmsa.co.za/view_exam.aspx?QualificationID=9.

We are keen to hear about how this series is assisting registrars and their supervisors in preparing for the FCFP (SA) examination. Please email us (corresponding author, M.N.) your feedback and suggestions.

## Multiple choice question: Single best answer

A 28-year-old human immunodeficiency virus (HIV)-positive non-pregnant woman presents one month after starting antiretroviral treatment (ART) for her clinic visit and cervical smear result at your rural district hospital. She is doing well on ART. The cytology from her cervical smear indicates that she has a high grade squamous intraepithelial lesion (HSIL). Colposcopy bookings are available in 6 months. What is the most appropriate next step?

- Perform a large loop excision of the transformation zone (LLETZ) under visual inspection of the cervix using acetic acid (VIA).
- Perform a VIA and biopsy
- Reassure her and repeat the test in 6 months
- Refer her for cryotherapy.
- Refer her or colposcopy

*Short answer: a*

### Expanded answer:

Cervical cancer is a national priority in South Africa as it is the second most common cancer reported in South Africa. In order to mitigate the impact of cervical cancer on health, South Africa implemented a comprehensive cervical cancer prevention and management programme. This utilised three strategies, namely, reducing oncogenic human papilloma virus (HPV) infections, detecting and treating cervical pre-cancer, and providing timely treatment and palliative care for invasive cancer.

Persistent infection of the cervix with oncogenic types of HPV types 16 and 18 causes cervical cancer. Human papilloma virus infection of the genital tract is mostly acquired by sexual contact with most people getting infected with HPV shortly after the onset of sexual activity. Infection with HPV is a prerequisite for the development of pre-invasive cervical intraepithelial neoplasia (CIN grades 1, 2, and 3), also known as low- and high-grade squamous intraepithelial lesions (LSIL and HSIL) (pre-cancer) and for progression to invasive cervical cancer.

There are various screening methods available for cervical cancer, as described in Table 1. Human papilloma virus DNA testing is recognised as the ideal cervical cancer screening method because of its high sensitivity, specificity, positive and negative predictive value, objectivity and reproducibility. However, the costs, logistics and technology requirements for successful implementation of current commercially available HPV testing are challenges for under-resourced health systems. Most clinics in the public healthcare in South Africa are using liquid-based cytology as compared with the traditional pap smear. All HIV-positive women are tested at diagnosis of HIV and then three yearly if their cytology is normal.

**TABLE 1:** Sensitivity and specificity of cervical screening methods.

Screening method	Sensitivity	Specificity
Visual inspection with acetic acid	69–77	82–87
HPV DNA testing	94–95	84–90
Cytology	70–84	88–95

*Source*: South African Department of Health. Cervical cancer prevention and control policy. Pretoria: South African National Department of Health; 2017

HPV, human papilloma virus; DNA, deoxyribonucleic acid.

The treatment of precancerous lesions is an essential part of secondary prevention and can either involve surgery (cryotherapy, CryoPen and thermal coagulation) or use excisional techniques, for example, LLETZ or LEEP (loop electrosurgical excisional procedure). The national guidelines recommend colposcopy and then LLETZ or cryotherapy; however, colposcopy services in many parts of the country are very limited and waiting times are long. Certain provinces, most notably KwaZulu-Natal, have embarked on a pragmatic approach by providing district hospitals with LLETZ machines and training staff on how to perform the procedure using the VIA. The sample removed is then sent for histology.

Large loop excision of the transformation zone with VIA before colposcopy has the following advantages:

- There is a decrease in waiting times.
- It allows the service to be decentralised.
- It prevents advanced cervical cancer.

The indications for LLETZ with VIA include the following:

- persistent low-grade squamous intraepithelial lesion
- atypical squamous cells of unknown significance (ASCUS)
- HSIL
- atypical squamous cells, cannot rule out high grade squamous intraepithelial lesion (ASC-H).

The following patients should be referred for colposcopy:

- atypical glandular cells
- adenocarcinoma *in situ*
- if malignant cells are present
- when benign endometrial cells are present
- if there is persistently no endocervical component
- large lesions evidenced with the VIA
- when micro-invasion is present
- patients with previous LLETZ/cone biopsy
- patients with bleeding disorders or on anticoagulants
- patients with structural, functional or electrical cardiac abnormalities
- pregnant patients
- if the lesion cannot be seen on VIA
- any case you are uncomfortable with.

Large loop excision of the transformation zone without colposcopy is a viable option in resource-constrained environments. It is a safe procedure in the hands of trained individuals and can be both diagnostic and therapeutic. One needs to choose the appropriate patient and refer when not comfortable. The main purpose is to decrease cancer of the cervix and prevent a delay in instituting definitive care.

#### Further reading

- South African Department of Health. Cervical cancer prevention and control policy. Pretoria: South African National Department of Health; 2017.
- KZN Department of Health. Guidelines for cervical screening and treatment to prevent cancer of the cervix. Pietermaritzburg, KZN Department of Health; 2018.
- Li ZG, Cen, JM, Di Chen G, Shu YH. Three-step versus ‘see-and-treat’ approach in women with high-grade squamous intraepithelial lesions in a low-resource country. Int J Gynecol Obstetr. 2009;106(3):202–205. https://doi.org/10.1016/j.ijgo.2009.04.011

### Short answer question: The family physician’s role as consultant and clinical governance leader

You are a family physician working in a rural sub-district, which contains a district hospital and several primary healthcare facilities and mobile clinic services. One of the nursing sisters from the mobile clinic service approaches you to discuss a 45-year-old female patient from one of the farms who missed her referral hospital colposcopy clinic appointment. Her routine Papanicolaou (pap) smear 18-months ago showed a high grade squamous intraepithelial lesion (HSIL). She is on antiretroviral therapy (first-line fixed dose combination therapy), and her viral load at the start of 2020 was 4350 copies per millilitre. It appears that there are other patients who have also missed their appointments at the referral hospital:

1. Based on the information given, what is your comprehensive three stage assessment of this patient? (3 marks)
2. What else would you like to explore on the history and examination of this patient? (5 marks)
3. Describe the comprehensive approach required to address this individual patient’s health needs. (5 marks)
4. Describe the possible root causes why these patients may have missed their appointments. (4 marks)
5. List the key steps of the quality improvement cycle you will implement to address this issue of missed appointments. (3 marks)

Total: 20 marks

#### Suggested answers

1. Based on the information given, what is your three-stage assessment? (3 marks)Provide a three-stage assessment:

- **Clinical**: (1)
  ■ High-grade squamous intraepithelial lesion requiring colposcopy
  ■ HIV-positive with an unsuppressed viral load on first-line antiretroviral therapy
  ■ Family planning unknown at this stage
- **Individual:** (1)
  ■ Fear and concerns unknown at this stage
- **Contextual:** (1)
  ■ Home situation unknown at this stage
  ■ Employment status and financial support source unknown at this stage

2. What else would you like to explore on the history and examination of this patient? (5 marks)

- History
  ■ General history
    ◦ Any constitutional symptoms such as weight loss and fever
    ◦ Any new symptoms, such as respiratory or abdominal systems, which may indicate either opportunistic infections or progression of her cervical lesion.
    ◦ Explore treatment adherence, which includes enquiring about medication side-effects and performing a mental health screen (especially depression).
    ◦ Risk factors, including smoking.
    ◦ Any history of procedures or surgical interventions, including sedation and anaesthesia.
- Women’s health history
    ◦ Previous pap smear history.
    ◦ Any new symptoms related to her reproductive system (vaginal discharge, abnormal vaginal bleeding and pelvic pain).
    ◦ Family planning history.
    ◦ Reproductive history (any previous pregnancies or miscarriages).
    ◦ Sexual health, including history of sexually transmitted infections.
- Individual
    ◦ Ideas around the results of her pap smear and reason for referral to the colposcopy service.
    ◦ Fears and concerns about her test results as well as her viral load control.
    ◦ Her expectations regarding the next steps of her treatment plan.
    ◦ Any loss of function because of her health conditions.
- Contextual
  ■ More information on her family context, including the HIV-status of her partner and children.
  ■ Occupational history, the support of her employer, as well as source(s) of income.
- Examination
  ■ General examination, especially blood pressure, pallor, oedema and lymph node enlargement.
  ■ Abdominal examination, for pain or masses.
  ■ Perform bimanual palpation to check for pain when moving the cervix and to exclude pelvic masses.
  ■ Perform speculum examination to inspect for abnormalities of the cervix.
*Mark allocation: The candidate needs to provide answers as grouped under the categories above (general history, women’s health history, individual, contextual and examination). Adequate responses receive half a mark each.*

3. Describe the comprehensive approach required to address this individual patient’s health needs. (6 marks)
*Any of the following (or other adequate responses) for half a mark each:*

- General health
  ■ motivational interviewing or brief behavioural change counselling: smoking cessation (smoking increases the risk of cervical abnormalities), ART adherence, screen for alcohol or drug use
  ■ prevention and screening, including breast examination, weight and diet
  ■ family planning review.
- Women’s health
  ■ Review the need for repeating a pap smear urgently and or arranging a new colposcopy appointment, in consultation with a referral hospital specialist or in line with local referral protocols.
  ■ If you identify any lesion/mass/polyp/erosion/ulcer/sore on speculum examination, avoid cervical screening and instead refer the same week for colposcopy or biopsy.
  ■ If available, use liquid-based cytology (LBC) and human papillomavirus (HPV) DNA testing. If cytology is unavailable, use visual inspection with acetic acid (VIA).
  ■ Manage the patient according to the following results (ensure close follow-up):
    ◦ If cervical screen negative (normal smear, HPV DNA negative or not done) – this is unlikely in this patient, given the previous HSIL result 18-months ago: (1) Counsel the patients regarding the findings; (2) Repeat cervical screen after 3-years (HIV-positive patient).
    ◦ If cervical screen abnormal (abnormal screen and/or HPV DNA positive) – this is more likely in this patient, given the previous HSIL result 18-months ago: (1) Counsel the patients regarding the findings. (2) If a normal Pap smear/LBC/VIA but HPV DNA positive, explain to the client that she does not have cancer but needs referral for colposcopy as HPV can cause cancer. (3) If VIA is positive or HPV DNA positive for HPV types 16 and 18: refer for cryotherapy or large loop excision of the transformation zone (LLETZ). (4) If an abnormal Pap smear/LBC, and/or VIA is suspicious for cancer or HPV DNA positive for other HPV types: refer for colposcopy. (5) Repeat screening in 1–3 years according to colposcopy findings/management needed.
- HIV management
  ■ Screen for tuberculosis symptoms and review the clinical staging of her HIV illness.
  ■ Her last viral load was unsuppressed around 18-months ago (viral load ≥ 50 copies per millilitre):
    ◦ Check record of attendance and adherence to medication. Give adherence support. Encourage partner and family disclosure.
    ◦ Explore if the patient experiences any side-effects on her antiretroviral therapy. Also ask about alcohol/drug use and perform a mental health screen (stress/anxiety/depression).
    ◦ Review other medication use, including over-the-counter medications. Consider medication interactions.
    ◦ Repeat viral load. If repeat viral load is again ≥ 1000 copies per millilitre, repeat CD4 (cluster of differentiation 4): if less than 200, start cotrimoxazole preventive therapy. This will also indicate that the patient has virological failure if the patient is on efavirenz- or nevirapine-based first-line regimen and the viral load is ≥ 1000 copies per millilitre on two consecutive occasions. However, if the patient is on a regimen containing dolutegravir, virological failure is defined as a viral load ≥ 1000 on at least three occasions over the course of 2 years.

4. Describe possible root causes why these patients may have missed their appointments. **(4 marks)**
*The candidate could use a Fishbone or root cause analysis approach to identify and deconstruct the problem at hand into underlying contributors and issues. In this case, the question lends itself to factors which may result in rather than prevent missed appointments. Allocate 1 mark for the heading and 1 mark for an example under each heading (maximum 4-marks).*

- Health services and health worker factors
  ■ Issues of access to care, especially considering living in a rural area she is working on a farm and/or living on a farm with notoriously poor access to health care, with only mobile clinic coverage, which may have been de-escalated during the first 18-months of the coronavirus disease 2019 (COVID-19) pandemic. Similarly, semi-elective and elective services, such as a colposcopy service at the referral hospital, may also have been affected by the de-escalated health services to decongest health facilities (in the scenario description above, it is stated that there are other patients who have also missed their appointments at the referral hospital). Both in pre-pandemic and in current times, the availability of decentralised colposcopy services is an important factor, which influences access to cervical screening.
  ■ Health workers have been repurposed and diverted from routine services to support the pandemic response. This includes primary care workers who provide mobile clinic services, who were tasked with screening and contact tracing, as well as regional hospital specialist staff who were tasked to help cover the COVID-service, including high care and intensive care services.
- Patient factors
  ■ Knowledge and attitudes towards pap smears and adherence to cervical screening may be affected by issues of poor understanding of cervical screening.
  ■ Knowledge may be affected by education levels, as well as sources from which knowledge on cervical screening is drawn.
  ■ Patients may also not be aware of the different approaches required for cervical screening in HIV-positive women.
  ■ Ideas, fears and concerns may revolve around the fear of the procedure (pain, embarrassment or stigmatisation).
- Social circumstances factors
  ■ Cultural aspects and world views may affect attitudes towards cervical screening.
  ■ Finances and social support may affect the patient’s ability to leave their home and family (including children) to access services.
  ■ Employer support and willingness to allow the patient to take time off work to attend remote health services.

5. List the key steps of the quality improvement cycle you will implement to address this issue of missed appointments. (3 marks)

- Establish the improvement team. (1/2)
- Agree on evidence-based criteria and local performance levels in order to set target standards for performance that you will measure. (1/2)
- Collect data to measure the criteria using appropriate methods and analyse the data. (1/2)
- Provide feedback to the findings relevant to role players and facilitate reflection in order to make sense of the findings. (1/2)
- Plan ways to improve performance and implement. Follow up and support implementation. (1/2)
- Repeat the audit and feedback after a suitable interval to determine whether there is improvement in performance. (1/2)

#### Further reading

- Botha MH, Dreyer G. Guidelines for cervical cancer screening in South Africa. South Afr J Gynaecol Oncol. 2017;9(1):8–12.
- Practical Approach to Care Kit (PACK). Primary care guide for the adult. Cape Town: Knowledge Translation Unit, University of Cape Town; 2020 [cited 2021 Aug 18]: Available from: https://knowledgetranslation.co.za/pack/pack-adult/
- Mazaza S, Gunst C. Chapter 8: Leadership and clinical governance. In: Mash B, editor. The handbook of family medicine. 4th ed. Cape Town: Oxford University Press, 2017; p. 360–385.
- Godfrey MA, Mathenjwa S, Mayat N. Rural Zulu women’s knowledge of and attitudes towards Pap smears and adherence to cervical screening. Afr J Primary Health Care Fam Med. 2019;11(1):1–6. https://doi.org/10.4102/phcfm.v11i1.1994
- Maree JE, Moitse KA. Exploration of knowledge of cervical cancer and cervical cancer screening amongst HIV-positive women. Curationis. 2014;37(1):1–7. https://doi.org/10.4102/curationis.v37i1.1209
- Maimela G, Nene X, Mvundla N, et al. The impact of decentralising colposcopy services from tertiary-level to primary-level care in inner-city Johannesburg, South Africa: A before and after study. BMJ Open. 2019;9(3):e024726. https://doi.org/10.1136/bmjopen-2018-024726

### Critical appraisal of qualitative research

Read the accompanying article carefully and then answer the following questions (*total 40 marks*). As far as possible, use your own words. Do not copy out chunks from the article. Be guided by the allocation of marks with respect to the length of your responses.

- Norris SA, Dickson LM, Buchmann EJ. Women’s accounts of the gestational diabetes experience – A South African perspective. S Afr J Obstetr Gynaecol. 2020;26(1):22–28.

#### 1. What was the research question of the study? (2 marks)

What were the experiences of women with gestational diabetes, as well as their coping strategies, challenges and health decisions?

#### 2. Identify three distinct arguments made by the authors to justify and provide a rationale for the study: (5 marks)

- The authors refer to established evidence that intervention strategies for gestational diabetes mellitus (GDM) in higher income countries do not necessarily apply to lower-to-middle income countries or low-resource settings.
- The authors identify a paucity of evidence of GDM lifestyle interventions in low-resource settings like theirs.
- They argue that culturally appropriate education and advice on diet and diabetes have been shown elsewhere to be more effective, and that there is some evidence that there are culturally influenced differences in how women experience GDM.
- However, they argue that there is currently no research on the experiences of women with GDM in the authors’ current setting.
- As part of a larger study attempting to develop a South African-applicable/relevant national GDM screening and lifestyle intervention programme, the authors argue that the experiences of women with GDM should be considered and included.

#### 3. Explain why a qualitative research methodology may be most appropriate for this question. Comment on where and how a quantitative data collection methodology might still be applicable. (3 marks)

Often exploring illness experiences in a specific geographical area where not much is known about them lends itself to in-depth qualitative research. This type of data is rich because it is not always restricted to discreetly contained questions and allows for the emergence of new data on beliefs, attitudes and experiences. These are not always easy to capture using quantitative data collection methods. Qualitative research studies provide room for probing and exploring the data in different directions during the interviewing process.

Likert scale questionnaires using questions guided by the research findings on the subject from elsewhere can play a comparative role. For example, coping strategies could be categorised into nominal groups wherein participants could then be requested to select in a questionnaire and quantitative data revealed. Finally, the authors identify several broad areas (coping strategies, health decisions, challenges, etc.) wherein a well-designed quantitative study could be used to cover and subsequently guide direction for a further in-depth qualitative study (sequenced mixed methods approach).

#### 4. Were the participants well described? Justify your response (3 marks)

Participants’ age, obstetric and family history, standardised criteria for diagnosis of GDM and body mass index gave a clear profile. However, given the use of interpreters, the home languages represented in the group may have helped to give a fuller picture of the diversity of the group. This also applies to the inclusion of socio-economic proxy information like employment status.

#### 5. Comment on the comprehensiveness and appropriateness of the data collection and analysis (10 marks)

Focus groups were used to collect data. However, the authors do not provide any reasons why individual semi-structured interviews were not used instead of, or in addition to focus groups. Whilst there were two research assistants who helped with translations, there is no mention of which languages were represented in the group and whether the research assistants were trained in translations. It is not clear how the diabetes conversation map was used to facilitate discussion. It is not clear what a structured script is. Is this a summary of responses? In qualitative research, it is usual to transcribe verbatim what is said so as to ensure no information is lost. Was the structured script the basis for analysis as they later talk about transcriptions of the audio recorded FGDs. The authors also do not describe the theme and sub-theme analysis process in enough detail. Even when qualitative coding software is not used, the specific steps they conducted and how many researchers were involved during each step is a standard reporting benchmark of good quality qualitative studies. The methodology of such studies should be explained and detailed well enough to be reproducible to promote its trustworthiness or rigour.

There are different forms of thematic analysis (e.g. codebook reliability, codebook and reflexive), each with its own specific methodological steps, and this is not described in the study report in enough detail. This description should also be preceded by a clear indication of whether the study undertook a deductive, inductive, latent or even semantic overarching approach to the analysis, and why this leads to the type of thematic analysis method they chose. They might also have indicated why they chose thematic analysis over other pattern-based approaches and methodologies like Grounded theory, qualitative content analysis and discourse analysis. The authors only indicate that the selection of quotations that reflected the group was done based on their intensity, frequency and context. However, it seems that there is a deductive component as they have preconceptions about what they may find in the data based on the literature they have reviewed, the subtopics they mention in the research question and lastly, the fact that they acknowledge that one of the themes was ‘unexpected’.

There is also no clear indication that the data collection or analysis reached saturation of data content, concepts or theory. The only notable indication or triangulation is the validation of the thematic analysis by a second researcher, and the respondent validation or member checking that features when the results were presented to the respondents.

#### 6. Appraise the reflexivity of the study (researcher positionality) (3 marks)

The researchers’ qualifications, training backgrounds and roles are described in this study. However, they do not discuss or report on how their experiences, perspectives and even identities played a role in the interviewing process and data analysis. Their relationships with the participants, the role of language and language barriers and the researchers’ roles in the larger study were not discussed or covered. Another benchmark of good qualitative research reporting is a reflexivity section or report.

#### 7. Comment on the reporting of the findings. In other words, were the descriptions of the findings clear and easy to understand? (3 marks)

An understanding of the standard reporting formats is being assessed here, along with a basic understanding of the importance of data with the theme. Any appropriate example from the study to substantiate the answer will suffice.

The themes were generally presented with an appropriate preceding summary and extracts from the data (and its source group) to exemplify the theme that the authors outlined. However, some subthemes were not adequately corroborated by the data extracts that the authors assigned to them (e.g. disbelief). Finally, the description of Theme 6 as ‘An unexpected theme’ is unusual; the theme should have been named through better analysis of the sentiments (disbelief or fear?) or conceptualisations underpinning it.

#### 8. Were the researchers’ conclusions appropriate and consistent with the data? Comment on the acceptability of their conclusions (5 marks)

The researchers rightfully outline the limitations of focus groups and acknowledge that the groups’ views cannot be generalised to any other groups or populations. They claim the group’s homogeneity to the group based on geography, socio-economic class and the tertiary level of care and diagnosis. However, their use of the term black as a homogenous description is problematic and ignores the obvious language (and therefore ethnic and social) diversity, along with the pitfalls of using descriptions of racial categories (urban black African Women is a very diverse group) in research. Finally, the researchers do not demonstrate how their findings demonstrate (or not) features that differ substantially from findings in other contexts, the possibility of which is an underlying rationale for the study. They also do not clarify how their findings will be used or applied to the subsequent phases of the larger study to which it belongs. This was one of the stated reasons for undertaking the study.

#### 9. Discuss the value of these findings for your practice (6 marks)

Use of the READER approach may be helpful here.

For a family physician in the South African public sector, the consideration of local healthcare users’ illness experiences of gestational diabetes would be helpful, particularly when capacitating district-level teams with how to support and care for these users before and after referral to tertiary centres for ongoing antenatal care. This may be especially relevant for women with previous histories of GDM and who present initially to primary care centres for the subsequent pregnancies. In this sense, the topic is relevant. However, the educational value is not remarkable, given the similarities of the themes to those of other chronic illness experiences with which family physicians are extremely familiar. The applicability of the results to a district hospital and primary care clinic setting is also limited, given that the care of women with GDM in South Africa is generally in tertiary centres. However, where the shared care of these clients is a logistical necessity in certain settings, an awareness of the themes of the study by family physicians and their multidisciplinary teams may be helpful and assist with adjusting the processes of care provided. This could specifically mean avoiding the ambiguities of the diagnosis that were generated by the mixed messages about GDM some of the women encountered during their care. From a discrimination point of view, the study’s poor methodological rigour would make it very difficult to accept the findings as trustworthy, and therefore, usable in a district health setting.

##### Further reading

- Pather M. Evidence-based family medicine. In Mash B, editor. Handbook of family medicine. 4th ed. Cape Town: Oxford University Press, 2017; p. 430–453.
- MacAuley D. Reader: An acronym to aid critical reading by general practitioners. Br J Gen Pract. 1994;44(379):83–85.
- Ross A, Mash B. African primary care research: Reviewing the literature. Afr J Prim Heal Care. 2014;6(1):a584. https://doi.org/10.4102/phcfm.v6i1.584
- The Critical Appraisals Skills Programme (CASP). 2021. CASP checklists [homepage on the Internet]. [cited 2021 Aug 17]. Available from: https://casp-uk.net/casp-tools-checklists/
- Dickson LM, Buchmann EJ, Norris SA. Women’s accounts of the gestational diabetes experience – A South African perspective. S Afr J Obstet Gynaecol. 2020;26(1):1–7.
- Chandler CIR, Reynolds J, Palmer JJ, Hutchinson E. ACT consortium guidance: Qualitative methods for international health intervention research. 2013 [cited 2021 Aug 31];2008(2008):100. Available from: www.actconsortium.org/qualitativemethodsguidance
- Pope C. Qualitative research in health care: Analysing qualitative data. BMJ. 2000;320(7227):114–116. https://doi.org/10.1136/bmj.320.7227.114

### Objectively structured clinical examination scenario

**Objective:** This station tests the candidate’s ability to diagnose and manage incontinence in an elderly patient.

**Role player:** Elderly lady

#### Instructions to the candidate

The following patient presents at the outpatient department of your district hospital. Please consult with her and develop a comprehensive management plan.

#### Instructions to examiner

See the objective above.

This is an integrated consultation station, in which the candidate has 15 min to consult with the patient, and 10 min to record a comprehensive assessment and management plan.

Familiarise yourself with the assessor guidelines, which detail the required responses expected from the candidate.

**FIGURE 1:**
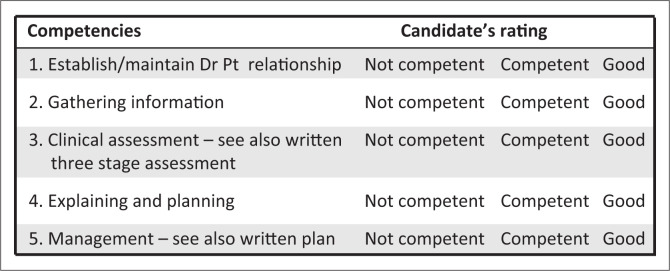
Candidate assessment sheet.

No marks are allocated. In the candidate assessment sheet (Figure 1), tick off one of the three responses for each of the competencies listed. Make sure you are clear on what the criteria are for judging a candidates’ competence in each area. When you have viewed the video, please access the PDF file with the comprehensive assessment and management plan.

The candidate should be given the clinical findings when the consultation commences if it is an online consultation, and when s/he asks for it, if directly observed.

#### Guidelines to examiner

For each domain, a candidate will be deemed competent if the task at hand is completed within the allotted time, and patient safety is preserved. When the task is incomplete, or patient safety is compromised, the candidate is deemed to be not competent in that domain.

#### Indicators:

- Establishing and maintaining the doctor-patient relationship: the competent candidate is respectful and polite, ensuring that the patient is always heard. Additionally, the good candidate displays empathy in eliciting the patient’s narrative and responds to the patient’s discomfort with compassion.
- Gathering information: the competent candidate gathers sufficient information (history and clinical findings) to make a working diagnosis (menopausal + stress incontinence). Additionally, the good candidate explores the psychosocial dimensions of the problem, probing the impact of the illness on this patient’s life. The good candidate should ask about chronic illness particularly diabetes and hypertension and any chronic meds – particularly diuretics
- Clinical assessment: the competent candidate makes a working diagnosis (menopausal + stress incontinence), and identifies the key psychosocial issues (isolation because of social embarrassment). Additionally, the good candidate makes a comprehensive three stage assessment (menopausal + stress incontinence + mild bladder prolapse; social isolation because of embarrassment; wants referral to the gynaecologist; concerns about sexual function).
- Explaining and planning: the competent candidate explains the assessment in simple, non-jargon language, and explores the patient’s preferences in managing this problem. Additionally, the good candidate offers the patient a few options, and facilitates decision-making by sharing information and encouraging autonomy.
- Management: the competent candidate applies an evidence-based management plan (consider hormone replacement therapy, refer to Gynae OPD, no pharmacological interventions for Stress Incontinence) including a safety net for red flags (infective symptoms; depression). Additionally, the good candidate considers local solutions (decreased tea intake, topical Estradiol, lubricant, pelvic floor exercises, insertion of vaginal ring), may address the sexual dysfunction, may suggest referral to a physiotherapist and discusses psychosocial interventions aimed at emotional well-being.

### Role player instructions

#### Personal circumstances

You are a married 63-year old lady, living with your husband. Your children live on their own, and you see them on weekends when they visit. You receive a private pension, as you were a teacher your whole life until retirement at age 60 years.

#### Main problem

Over the last few months, you have been wetting yourself. When you cough, sneeze or stand up, you pass urine unintentionally.

### Additional information (if asked)

- General health – good. You are not on any medication for any chronic illness
- Gynaecological history – you had three children, all normal births, normal pregnancies. Stopped your periods about 10 years ago. Never used hormone therapy. Had normal Pap smear 2 years ago.
- Lifestyle habits – non-smoker; occasionally have a glass of wine; one cup of coffee a day; lots of tea (7–8 cups/day) and very little water. Enjoy gardening (almost daily)
- Emotionally – generally ok, but very anxious about this problem, as it is affecting your life deeply.

### Concerns or worries or expectations (if asked)

Missing church, does not want to go for shopping anymore – embarrassing because of the smell of urine
Sexual intercourse has been painful for the last 2–3 years – very dry
Would like a referral to a specialist to solve this problem – you do not mind if you can have an operation!

### Clinical findings

General examination:

- Anxious elderly lady, body mass index (BMI) 22, neatly dressed.
- Vitals: Blood pressure (BP) 128/78; heart rate 67/min; random finger prick glucose 5.6 g/dL.

Systematic examination:

- Cardiorespiratory examination – normal
- Abdominal examination – normal
- U-dipstick – no abnormalities detected.
- Vaginal speculum examination: no bleeding; marked atrophy of vaginal mucosae; mild prolapse of bladder; cervix looks normal
- Vaginal examination: no masses detected; uterus not enlarged.

#### Further reading

- Department of Health, South Africa. Stress incontinence. In: Commerford PJC, Gebhardt GS, De Waal R, Maartens G, editors. Standard treatment guidelines and essential medicines list for SA. Hospital LEVEL, adults. 2019 ed. South African Department of Health. Hospital Level Standard Treatment Guidelines and Essential Medicines List. Pretoria: South African National Department of Health; 2019.

